# Farm animal genetic and genomic resources from an agroecological perspective

**DOI:** 10.3389/fgene.2015.00153

**Published:** 2015-04-30

**Authors:** Michèle Tixier-Boichard, Etienne Verrier, Xavier Rognon, Tatiana Zerjal

**Affiliations:** GABI, INRA, AgroParisTech, Université Paris-SaclayParis, France

**Keywords:** agroecology, ecosystem services, local breeds, genetic variation, genomics, animal breeding

Agroecology, as a scientific approach, relies on a better knowledge of biodiversity at all levels of organization and function, in order to better manage agricultural production systems, from farm scale to landscape. Ecological concepts such as functional redundancy, complementary use of resources, can be applied to farming systems, with the purpose of improving their resilience. Transposing the concepts of agroecology to livestock production has been recently proposed by Dumont et al. (2013). One of the principles proposed for the design of sustainable animal production systems is to enhance diversity within animal production systems in order to strengthen their resilience. Why is it so? An increased biodiversity allows benefiting from complementary aptitudes. For example, in the case of disease resistance, the diversity of hosts will limit the risk of the specialization of a highly pathogenic agent with devastating consequences. It does not mean that diseases will not occur but the spread of infections and the overall impact on animal health should be limited (Springbett et al., 2003).

How the agroecology concepts can be applied to farm animal genetic resources and how the genomics approach may be used to facilitate it?

Biodiversity in livestock production systems may be considered at all scales, from individuals and breeds to species and ecosystems. Several levels may be considered to increase biodiversity in livestock production systems, such as intermixing species within production systems. This paper will focus on within-species biodiversity that goes from local breeds to highly selected populations. At the population scale, one possibility is to increase the number of breeds in use, or to produce new composite populations, as done for the Creole cattle in the French West Indies (Gautier and Naves, 2011). At the local scale (i.e., the farm), the increase of biodiversity may be obtained by intermixing breeds, by using crossbreeding but also by monitoring individual genetic diversity within a group. What could be the drivers for such a trend toward increased biodiversity? Stratified crossbreeding between local breeds is likely to increase the level of diversity at the individual level, because of a higher heterozygosity of F1, but may not increase the diversity within a group since F1 animals are likely to inherit the most frequent alleles present in their parental breeds. The main issue is still to maintain genetic diversity within each parental breed. Breeding for an increased production level has led to a decrease of within-breed variability (Danchin-Burge et al., 2012) although measures have been taken to limit this decrease. Actually, another incentive than selection response is needed to trigger an increased use of biodiversity in livestock systems.

We propose to use the conceptual framework of ecosystem services for this purpose. Ecosystem services are benefits that human populations get from natural or managed ecosystems. This framework includes not only the provision of food but also environmental and socio-cultural services. Although the concept of ecosystem services is still evolving and open to debate (Lele et al., 2013), it is applied to value ecosystem services in complex ecosystems incorporating livestock, as shown by Silvestri et al. (2013) in Kenya. The need to quantify various services and their interactions is opening a new research field, which is relevant to characterize genetic resources in various production systems. Moreover, the frame of ecosystem services is strongly connected with sustainability, as shown by Broom et al. (2013) for sylvo-pastoral systems. However, it should not be restrained to the extensive systems but should involve all production systems with the genetic resources embedded in them.

The framework of ecosystem services requires the development of a multi-criteria assessment of the added value of an individual or a breed, beyond its contribution to food production, which is generally valued by a market price. Regarding local breeds, the production of high quality food for niche markets has often been considered as a very good opportunity for their preservation, besides the development of commercial breeds (Lambert-Derkimba et al., 2013). In addition to distinctive products, local breeds may also provide environmental services, which are still difficult to assess and value. Generally, such niche markets remain fragile, and the cultural services they provide are difficult to quantify. Environmental services may be positive or negative, local or distant. For example, positive services may involve fire prevention through extensive grazing, maintenance of open landscapes or habitats for wild species, manure production as a fertilizer for crops. On the opposite, negative services may be the increased pollution due to excess of manure, at a local scale, or the increased deforestation for production of soy-bean or maize, at a distant scale. Socio-cultural services may involve rural development, landscape management, ecotourism, which valuation is a real challenge. Raising livestock is an activity embedded into an agroecosystem, combining livestock and plant production benefits from complementarity in the use of resources.

Research is needed to develop methods and indicators to quantify different types of services in order to integrate them into breeding goals. There will be trade-offs between services, as well as between productive and adaptive traits in livestock. Quantitative genetics has tools to combine different traits in a breeding goal, whatever the correlation between traits, and could do the same to combine different services provided that appropriate weighting parameters are defined. Stratified crossbreeding is another approach to combine different aptitudes in F1 animals in order to optimize trade-offs between traits. The possibility to apply this approach to other services than production remains to be investigated.

The identification of traits or functions which are the basis for these services is an important research issue where genomics can play a major role. The genome is an archive of the population past and recent history but, until recently, few markers were available to read this archive. In the last 10 years, whole genome sequencing tools have developed allowing to understand population history and to unravel the genetic determinism of complex traits. Thus, genome sequencing has provided a universal frame for all geneticists working on a species, to share tools and data. At the same time conservation genetics is moving toward conservation genomics (Ouborg et al., 2010). High density SNP chips are powerful tools to monitor genetic diversity at different scales: animals within a herd, herds within a landscape and breeds within a species. In terms of functional diversity, sequencing can be used to reveal the footprints of natural or artificial selection, provided that relevant statistical methods are used to distinguish these footprints from drift effects. For example, landscape genomics may help to identify the genetic basis for some environmental services, such as climatic adaptation (e.g., Flori et al., 2012), tolerance to rough diets, or resistance to pathogens, provided that data are available to document these services. At present, genomic selection has revolutionized the organization of cattle selection and it is likely to be applied in other species, provided that a sufficiently large reference population is established to ensure reliable associations between genotype and phenotype. Such data sets already exist, as shown by Hayes et al. (2009) who studied adaptation to tropical climate of Holstein Friesian in Australia.

Genomics can support an agroecological management of animal genetic resources at three levels.

The first one is the monitoring of genetic diversity at any scale, and more particularly at the herd level: genotyping animals within a herd will allow to calculate the mean genetic distance of any external animal, sire, or dam, to the herd, and to integrate the benefit associated with the introduction of an external animal to the herd diversity. This procedure is similar to those used to minimize inbreeding in a mating plan at a breed level, but it takes place at the herd level, to maximize its diversity. This will require the reduction of costs for genotyping. As an example, the low density (LD) cattle chip is the most used and the cheapest SNP array. Other requirements are that breeders have easy access to the genotyping results, through their breeding organization or technical services, and to guidelines explaining how to use these results to monitor the diversity within herds. The benefit is to maximize genome diversity and the overall genetic resource within the herd. Research is needed to prove that maximizing genetic diversity will indeed improve the tolerance of the herd to climate change and extreme events which are likely to become more frequent, and guarantee a stable performance of the group, which is an important objective for the farmer. Genomic selection and individual dairy cow genotyping are producing large datasets suitable to test such a relationship between genetic diversity and herd resilience to extreme events. This would represent a change in breeding methods, since the best herd will not be an ensemble of the best animals but the best set of diverse animals.

The second one is the association of genomic regions with a range of phenotypes or performance levels. Monitoring these regions may provide a tool to tailor the genetic make-up of a herd with regards to specific traits that need to be combined at the herd level. This approach targets specific aptitudes to be combined, going beyond the first approach which aimed at verifying that the herd harbors a sufficiently high diversity, leaving options for future choices. The main challenge is to define the relevant phenotype to be predicted with molecular markers. Which phenotypes correspond to environmental or socio-cultural services? Research is needed on these issues, in close connection with the farmers, who are willing to better characterize their breed and raise awareness about its value, and with other actors who are benefiting from the service. Genomic regions associated to a desired trait or service should be validated at a large scale so that their effect is likely to be observed in any herd or any herd x environment combination. However, environmental services may depend on the herd location and be difficult to assess on a large scale because of genotype by environment interactions (GxE). In this case the farmer may choose a specific genetic profile for its herd.

The third one is targeting local breeds, where genomic selection and large scale reference populations are not available. There are two main issues for these breeds: managing their genetic variability and characterizing their specific features. Such breeds harbor original characteristics, either for product quality, or adaptation to specific environments, but the genetic basis for these characteristics is seldom known. If farmers had access to this information, how would they use it? What if adaptive features involved epigenetic changes that cannot be tested by standard SNP genotyping? Research is obviously needed in this regard. The use of such information may require a change of scale in the breed management, to record more traits and share more data between herds. This is more an organizational challenge and a social issue than a genetic issue. Furthermore, the identification of original features in local breeds may trigger the onset of crossbreeding programs to introduce such features in commercial populations, which raises strong issue for benefit sharing and for preserving breed specificities. Implementing genomics for the management of genetic variability appears relatively easy, provided that the genotyping cost is affordable, which is not so obvious for small populations. To share costs, a multi-breed genotyping tools could be an option to explore. With this approach, farmers will get accurate information about drift, population fragmentation or introgression events affecting a breed. Population fragmentation is typically expected when small size herds do not exchange animals, to a point that the genetic difference can be very high among them. Such a situation with a low within-herd variability but a high between herds variability could be favorable at the breed level from the viewpoint of agroecology (diversity and complementarity between herds) but it might question the breed definition and the breed identity. The same issue could appear if introgression events are detected: the breed would not anymore be the “pure” local breed as often described, even though this introgression could contribute to the breed evolution and facilitate its adaptation to future conditions. It is not to be excluded that the genomic characterization will enhance social issues, revealing how farmers are managing their breed.

In conclusion, setting the management of animal genetic resources in an agroecological perspective raises two major issues where research is needed: the possible transposition of ecosystem services to animal breeding and the impact of genomic tools on the breed concept and the management of genetic diversity at different scales.

## Conflict of interest statement

The authors declare that the research was conducted in the absence of any commercial or financial relationships that could be construed as a potential conflict of interest.
